# Ginsenoside Rh1 Alleviates HK-2 Apoptosis by Inhibiting ROS and the JNK/p53 Pathways

**DOI:** 10.1155/2020/3401067

**Published:** 2020-07-06

**Authors:** Qi Yang, Lin Qian, Song Zhang

**Affiliations:** ^1^Chengdu University of Traditional Chinese Medicine, Chengdu, China; ^2^Hospital of Chengdu University of Traditional Chinese Medicine, Chengdu, China

## Abstract

**Background:**

Cisplatin is widely used in the treatment of malignant patients; however, its adverse nephrotoxic effects limit its clinical use. Ginsenoside Rh1 is a main component of ginseng and has many pharmaceutical effects, including immunomodulatory effects.

**Objective:**

The objective of this research is to assess the effects of ginsenoside Rh1 on a cisplatin-induced HK-2 injury model and to study its potential effect mechanisms.

**Methods:**

HK-2 cell vitality was assessed via Cell Counting Kit-8 (CCK-8) assay. Carboxyfluorescein succinimidyl ester/propidium iodide (CFSF/PI) staining was used to detect the apoptosis of HK-2 cells. ROS expression was detected by DCFDA. The expressions of JNK, p53, caspase-3, Bax, and NGAL were detected by western blot.

**Results:**

Ginsenoside Rh1 was found to increase the vitality of HK-2 cells and inhibit ROS production and the apoptosis of HK-2 cells in a cisplatin-induced injury model. Ginsenoside Rh1 was found to inhibit the expression of JNK, p53, caspase-3, Bax, and NGAL in a cisplatin-induced injury model.

**Conclusion:**

Ginsenoside Rh1 alleviated HK-2 apoptosis in a cisplatin-induced injury model by inhibiting ROS production and the JNK/p53 pathway. Ginsenoside Rh1 may be a promising drug for the alleviation of cisplatin-induced nephrotoxicity in malignant patients.

## 1. Introduction

Cisplatin is one of the most common drugs that is used in the treatment of cancers; however, its nephrotoxic side effects severely restrict its clinical use. It has been demonstrated that pediatric patients who receive cisplatin treatment have a greater than 70% chance of developing kidney dysfunction [1], and patients have been reported to suffer from kidney damage several days after receiving cisplatin treatment [2]. Successive cisplatin treatment can cause progressive and permanent loss of kidney function, even if preventive measures are taken [3]. Several studies have shown that cisplatin-induced nephrotoxicity is closely associated with excessive reactive oxygen species (ROS) generation [4]. The accumulation of ROS induces apoptosis signal transduction and activates apoptotic proteins, such as caspase-3 and Bax, and causes apoptosis [5, 6]. Previous research has proven that the inhibition of ROS generation could inhibit cisplatin-induced apoptosis [7].

The JNK and p53 cellular signaling pathways have been demonstrated to play an important role in cisplatin-induced nephrotoxicity. Previous studies have shown that the activation of the JNK and p53 pathways by cisplatin can cause cellular apoptosis via the activation of apoptotic molecules. Moreover, the inhibition of the expression of JNK or p53 has been found to improve cisplatin-induced apoptosis [7, 8]; therefore, this strategy may be effective in the alleviation of cisplatin-induced nephrotoxicity.

Many traditional Chinese medicine (TCM) products have been used in the treatment of renal injury. Renal fibrosis has been found to be improved by TCM products, including resveratrol, curcumin, berberine, and poricoic acid, among others [9–12]. TCM products can also improve acute kidney injury by inhibiting inflammatory response and proapoptotic transcription factors [13]. According to previous research, TCM products have the potential to alleviate kidney damage. Ginseng is a TCM product that has been used for thousands of years to improve the human physical condition and is distributed worldwide. Ginsenoside Rh1 has been demonstrated to be the main component of ginseng [14] and to have many pharmacological effects. Jung et al. found that ginsenoside Rh1 can significantly inhibit ROS production and the expression of proinflammatory molecules in BV2 cells induced by lipopolysaccharide [15]. Ginsenoside Rh1 also has been demonstrated to have an immunomodulatory effect on inflammation by affecting the expression of cytokines and some pathways [16]. According to previous research, it is speculated that ginsenoside Rh1 might be able to alleviate cisplatin-induced nephrotoxicity by suppressing ROS generation and its downstream pathways.

In this research, HK-2 (human renal tubular epithelial) cells were used as the experimental cells. Cisplatin was used to induce a nephrotoxicity model. It was found that ginsenoside Rh1 can attenuate cisplatin-induced apoptosis by inhibiting ROS production and the JNK/p53 pathways.

## 2. Materials and Methods

### 2.1. Cell Culture and Treatment

HK-2, a human kidney tubular cell line, was purchased from Shanghai Biotechnology Company (Shanghai, China). HK-2 cells were cultured in Dulbecco's modified Eagle's medium with 10% fetal bovine serum, streptomycin (100 *μ*g/ml), and penicillin (100 IU/ml) purchased from Thermo Fisher Scientific (Waltham, MA, US). Trypsin was purchased from Sigma-Aldrich (St. Louis, Missouri, USA) and used for subculture every 2–3 d. HK-2 cells were transmitted once. In the ginsenoside Rh1 experiment, HK-2 cells were divided into a control group, cisplatin group, and ginsenoside Rh1 + cisplatin group. Ginsenoside Rh1 was added to culture plates 30 min before incubation with cisplatin. The doses of ginsenoside Rh1 and cisplatin used in the experiments were as follows. In the ROS inhibitor experiment, HK-2 cells were divided into three groups, namely, the control group, cisplatin group, and 10 *μ*M acetylcysteine + cisplatin group. Acetylcysteine was added to culture plates 30 min before incubation with cisplatin. In the anisomycin experiment, HK-2 cells were divided into three groups, namely, the control group, cisplatin group, and anisomycin + ginsenoside Rh1 + cisplatin group. Anisomycin and ginsenoside Rh1 were, respectively, added to culture plates 1 h and 30 min, before incubation with cisplatin. HK-2 cells were collected 24 h after adding the cisplatin to the culture plates.

### 2.2. Drugs and Reagents

Ginsenoside Rh1 and cisplatin were purchased from Chengdu Desite Biotechnological Company (Chengdu, Sichuan, China). Anisomycin was purchased from R & D Company. Cell Counting Kit-8 (CCK-8), carboxyfluorescein succinimidyl ester (CFSE), and propidium iodide (PI) were purchased from Sigma-Aldrich (St. Louis, Missouri, USA). DMEM and fetal bovine serum were purchased from Gibco (Carlsbad, CA, USA). A DCFDA-Cellular ROS Assay Kit and Hoechst stains were purchased from Abcam (Cambridge, MA, USA), as were antibodies including p53, JNK, caspase-3, Bax, and neutrophil gelatinase-associated lipocalin (NGAL). Finally, acetylcysteine, a ROS inhibitor, was also purchased from Abcam (Cambridge, MA, USA).

### 2.3. Cell Vitality Assay

The vitality of HK-2 cells was assessed via CCK-8 agent. HK-2 cells were cultured in 96-well plates to 60–70% confluency. After treating cells with cisplatin for 24 h, CCK-8 working solution was added to each well for 1 hour. Ginsenoside Rh1 was then added to the 96-well plates 30 min before incubation with cisplatin for 24 h, after which CCK-8 working solution was added. A microplate reader (Thermo Fisher Scientific, Waltham, MA, USA) with absorbance at 450 nm was used to read the optical density values.

### 2.4. CFSE/PI Staining Assay

Apoptosis was assessed by CFSE/PI staining. HK-2 cells were cultured in 24-well plates to 60–70% confluency. At the indicated time, the HK-2 cells were incubated with 200 *μ*l of 5 *μ*M CFSE working liquid for about 30 min. After CFSE staining, the CFSE stain was removed, and the cells were gently washed with phosphate-buffered saline (PBS). Then, 200 *μ*l of PI working solution was added to each well for 5 min, followed by gentle washing with PBS. Images were immediately taken under a fluorescence microscope (Olympus, Tokyo, Japan). Viable cells emitted green light and apoptotic cells emitted red light under a fluorescence microscope. The average fluorescence intensity of the apoptotic cells was analyzed by Image-Pro Plus software.

### 2.5. ROS Staining

ROS expression was detected by DCFDA. HK-2 cells were cultured in 24-well plates. At the indicated time, the cells were stained with 200 *μ*l of 20 *μ*M DCFDA for 30 min at 37°C and gently washed with PBS. Then, 200 *μ*l of Hoechst working solution was added to each well to stain the nuclei for 5 min, followed by gentle washing with PBS. Images were immediately taken under a fluorescence microscope. The average fluorescence intensity of the apoptotic cells was analyzed by Image-Pro Plus software.

### 2.6. Western Blot

The extraction of HK-2 cell proteins and the western blot protocol were performed as reported in the previous research [17–19]. Sodium dodecyl sulfate-polyacrylamide gel electrophoresis (SDS-PAGE) was used to separate proteins, which were then transferred to polyvinylidene difluoride (PVDF) membranes. The membranes were blocked with 5% milk. After blocking, the membranes were incubated with diluted primary antibodies overnight at 4°C according to the manufacturer's instructions. After washing with PBST containing 0.1% Tween-20 PBS solution for 5 min three times, the secondary antibody was incubated for 1 h at room temperature. *β*-actin was used as an internal control. The relative expression levels of JNK, p53, caspase-3, Bax, and NGAL were calculated by their ratios to *β*-actin.

## 3. Statistical Analysis

The data are reported as the mean ± standard deviation. The results of the two groups were compared by two independent *t*-tests. All statistical analyses were conducted by SPSS 22.0, and a statistically significant difference was defined as *P* < 0.05.

## 4. Results

### 4.1. Cisplatin Inhibited HK-2 Cell Vitality

**Figure 1:**
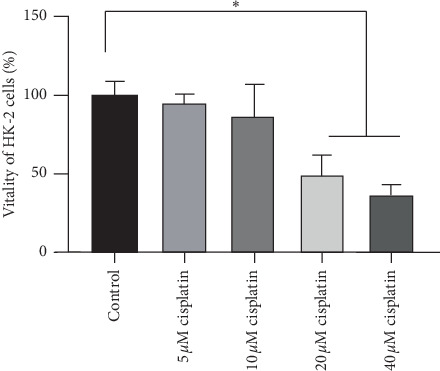
Cisplatin decreased the vitality of HK-2 cells. ^*∗*^*P* < 0.05 vs. all other groups.

The vitality of the HK-2 cells was inhibited after treatment with cisplatin for 24 h, as assessed via CCK-8 assay. 20 *μ*M cisplatin significantly inhibited the vitality of HK-2 cells compared with the control group, as presented in Figure 1. Therefore, a dose of 20 *μ*M cisplatin was used in the subsequent experiments.

### 4.2. Ginsenoside Rh1 Increased HK-2 Cell Vitality

**Figure 2:**
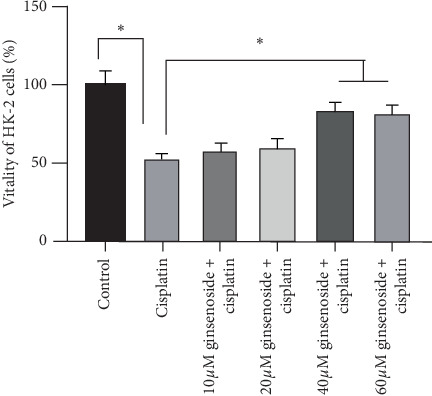
Ginsenoside Rh1 increased the vitality of HK-2 cells in a cisplatin-induced injury model. ^*∗*^*P* < 0.05 vs. all other groups.

Ginsenoside Rh1 increased the vitality of the HK-2 cells in a cisplatin-induced HK-2 injury model. 40 *μ*M ginsenoside Rh1 significantly increased the vitality of HK-2 cells compared with the cisplatin group, as presented in Figure 2. Therefore, a dose of 40 *μ*M ginsenoside Rh1 was used in the subsequent experiments.

### 4.3. Ginsenoside Rh1 Inhibited the Apoptosis of HK-2 Cells

**Figure 3:**
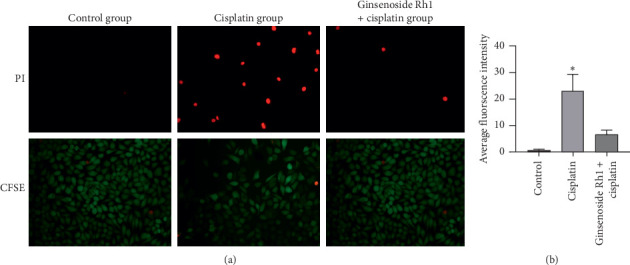
Ginsenoside Rh1 improved the apoptosis of HK-2 cells in a cisplatin-induced injury model. (a). Representative fluorescence images of the apoptosis of HK-2 cells; (b) quantitative fluorescence intensities of PI staining. ^*∗*^*P* < 0.05 vs. all other groups.

To confirm the protective effect of ginsenoside Rh1 on the inhibition of the apoptosis of HK-2 cells, apoptosis cells were detected by PI and CFSE. Cisplatin significantly increased the apoptosis of HK-2 cells compared with the control group; however, ginsenoside Rh1 significantly decreased the apoptosis of HK-2 cells compared with the cisplatin group, as presented in Figure 3. The results demonstrate that ginsenoside Rh1 has a protective effect in cisplatin-induced HK-2 cell apoptosis.

### 4.4. Ginsenoside Rh1 Inhibited ROS Production

**Figure 4:**
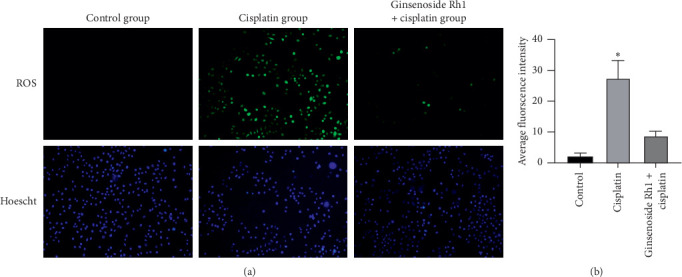
Ginsenoside Rh1 inhibited ROS production in a cisplatin-induced injury model. (a) Representative fluorescence images of ROS; (b) quantitative fluorescence intensities of ROS staining. ^*∗*^*P* < 0.05 vs. all other groups.

Cisplatin significantly increased ROS production in HK-2 cells compared with the control group; however, ginsenoside Rh1 significantly decreased ROS production in the HK-2 cells compared with the cisplatin group, as presented in Figure 4. The results demonstrate that ginsenoside Rh1 can inhibit cisplatin-induced ROS generation.

### 4.5. Ginsenoside Rh1 Inhibited Apoptotic and Kidney Injury Protein Expression

**Figure 5:**
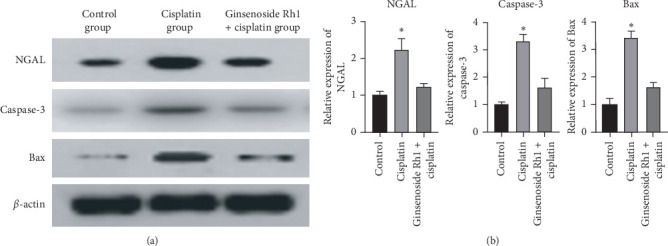
Ginsenoside Rh1 inhibited the expression of apoptotic proteins and kidney injury protein. (a) Representative bands of NGAL, caspase-3, and Bax; (b) summarized data showing the band intensity ratios to *β*-actin normalized to the values in the control group. ^*∗*^*P* < 0.05 vs. all other groups.

Cisplatin significantly increased the expression of apoptotic proteins caspase-3 and Bax, as well as the expression of the kidney injury protein NGAL, compared with the control group. Ginsenoside Rh1 inhibited the expression of caspase-3, Bax, and NGAL, as presented in Figure 5.

### 4.6. Ginsenoside Rh1 Inhibited JNK and p53 Expression

**Figure 6:**
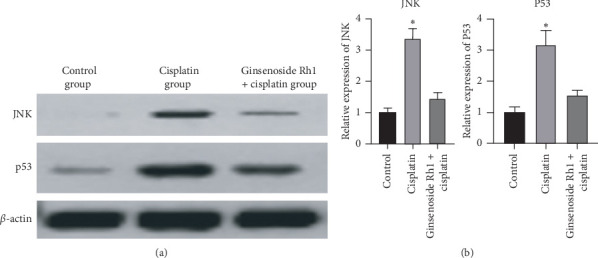
Ginsenoside Rh1 inhibited the expression of JNK and p53. (a) Representative bands of JNK and p53; (b) summarized data showing the band intensity ratios to *β*-actin normalized to the values in the control group. ^*∗*^*P* < 0.05 vs. all other groups.

Cisplatin significantly increased JNK and p53 expression compared with the control group; however, ginsenoside Rh1 significantly decreased JNK and p53 expression compared with the cisplatin group, as presented in Figure 6.

### 4.7. ROS Inhibitor Inhibited JNK Expression

**Figure 7:**
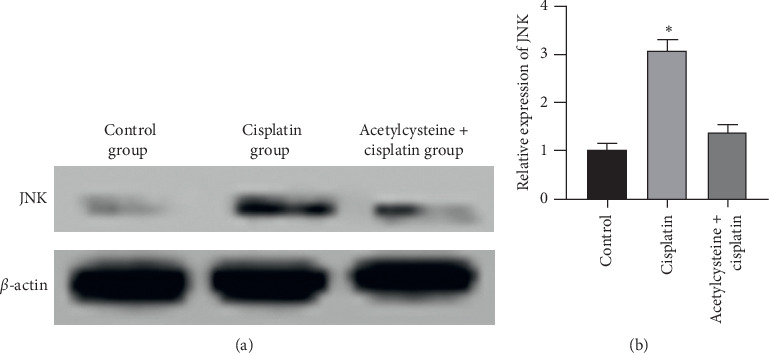
ROS inhibitor inhibited JNK expression. (a) Representative band of JNK; (b) summarized data showing the band intensity ratios to *β*-actin normalized to the values in the control group. ^*∗*^*P* < 0.05 vs. all other groups.

Cisplatin significantly increased JNK expression compared with the control group; however, the ROS inhibitor significantly decreased JNK expression compared with the cisplatin group, as presented in Figure 7. Therefore, it is speculated that ginsenoside Rh1 inhibits HK-2 cell apoptosis via the ROS/JNK pathway.

### 4.8. JNK Agonist Reversed the Expression of p53 Inhibited by Ginsenoside Rh1

**Figure 8:**
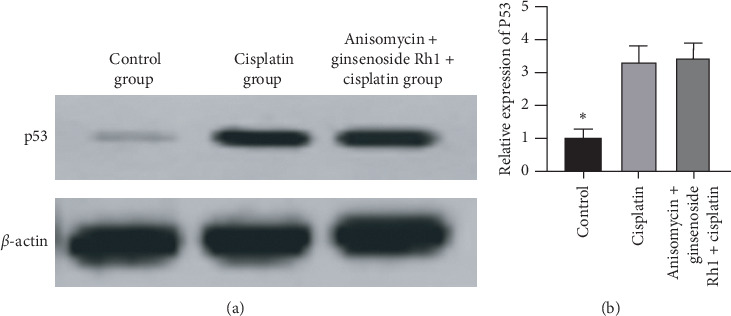
JNK agonist reversed p53 expression. (a) Representative band of p53; (b) summarized data showing the band intensity ratios to *β*-actin normalized to the values in the control group. ^*∗*^*P* < 0.05 vs. all other groups.

To confirm the relationship between JNK and p53, a JNK agonist was used in the experiment. Cisplatin significantly increased p53 expression compared with the control group. Moreover, there was no difference between the cisplatin group and the anisomycin + ginsenoside Rh1 + cisplatin group, indicating that anisomycin can reverse the expression of p53 inhibited by ginsenoside Rh1, as presented in Figure 8.

### 4.9. JNK Agonist Reversed the Expression of Apoptotic and Kidney Injury Protein Inhibited by Ginsenoside Rh1

**Figure 9:**
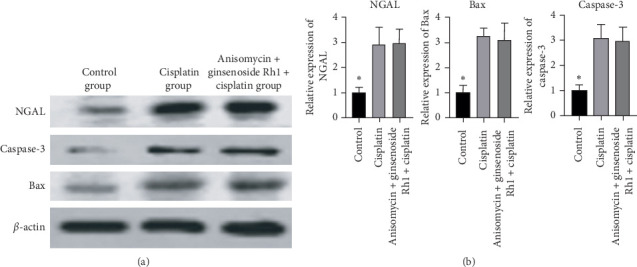
JNK agonist reversed protection by ginsenoside Rh1. (a) Representative bands of NGAL, caspase-3, and Bax; (b) summarized data showing the band intensity ratios to *β*-actin normalized to the values in the control group. ^*∗*^*P* < 0.05 vs. all other groups.

Cisplatin significantly increased the expression of caspase-3, Bax, and NGAL. There was no difference between the cisplatin group and the anisomycin + ginsenoside Rh1 + cisplatin group, indicating that anisomycin can reverse the effect of ginsenoside Rh1 inhibiting the expression of caspase-3, Bax, and NGAL. Therefore, it is speculated that ginsenoside Rh1 may inhibit HK-2 cell apoptosis via the JNK/p53 pathway, as presented in Figure 9.

## 5. Discussion

The following new findings were made in the present research: (1) ginsenoside Rh1 improved the vitality of HK-2 cells; (2) ginsenoside Rh1 inhibited HK-2 cell apoptosis; (3) ginsenoside Rh1 inhibited excessive ROS production; (4) ginsenoside Rh1 inhibited the expression of apoptotic proteins; (5) ginsenoside Rh1 inhibited HK-2 cell apoptosis, potentially via the ROS/JNK/p53 pathway.

Cisplatin is an effective chemotherapy drug used for the treatment of many different solid tumors; however, cisplatin-induced nephrotoxicity is one of its most significant adverse effects [20]. The maximal accumulation of cisplatin occurs in proximal tubules of the kidney, which experience the most severe cell death and inflammation [21]. Cisplatin-induced renal injury is associated with renal tubular cell apoptosis and tissue injury and leads to kidney dysfunction. Therefore, in this research, human kidney tubular cells were chosen as experimental cells. The morphological analysis of cisplatin-induced nephrotoxicity has indicated that apoptosis and necrosis are common cellular injuries to the renal tubules. Therefore, the inhibition of the apoptosis of renal tubular cells can improve kidney function in cisplatin-induced nephrotoxicity. Excessive ROS production induced by cisplatin has been proven to be one of the mechanisms that cause tubular cell apoptosis. In the present study, it was found that ginsenoside Rh1 reduced the cisplatin-induced overproduction of ROS in HK-2 cells. One of the mechanisms by which ginsenoside Rh1 alleviates the apoptosis of HK-2 cells might be the inhibition of ROS production.

Apoptosis plays an important role in maintaining the stability of internal environments. However, excessive apoptosis has adverse effects on tissues. Kidney injury can be caused by renal tubular apoptosis induced by cisplatin. JNK and p53 are important molecules involved in cisplatin-induced nephrotoxicity via the induction of cellular DNA damage, cell cycle arrest, and ultimately apoptosis [22]. The inhibition of JNK has been proven to alleviate cisplatin-induced nephrotoxicity [23]. In the present study, it was found that the inhibition of JNK expression by ginsenoside Rh1 decreased the expression of apoptotic genes. Moreover, p53, a tumor suppressor protein, also plays an important role in cisplatin-induced nephrotoxicity. Cisplatin upregulates the expression of p53, resulting in cell cycle arrest at the G1 phase and the activation of apoptotic proteins such as caspase-3 and Bax [24, 25]. Cummings et al. have demonstrated that the inhibition of p53 alleviates cisplatin-induced apoptosis in renal cells [5]. In an in vivo study, Molitoris et al. also confirmed the p53-dependency of cisplatin-induced nephrotoxicity [26]. The findings of this research are consistent with the results of the present study, in which it was found that ginsenoside Rh1 improved the apoptosis of HK-2 by inhibiting JNK and p53. Moreover, the results demonstrate that the inhibition of ROS can reduce the expression of JNK, which proves that ROS can activate JNK in HK-2 cells.

Ginsenoside Rh1 has an antioxidant effect by decreasing ROS production stimulated by H_2_O_2_ and xanthine-xanthine oxidase [27]; this is similar to the results of the present study, in which it was found that ginsenoside Rh1 inhibits the production of ROS stimulated by cisplatin. Ginsenoside Rh1 has also been proven to suppress JNK expression in U87 MG cells [3]. In the present study, it was found that ginsenoside Rh1 can inhibit not only JNK expression but also p53 expression. To investigate the relationship between JNK and p53 in a cisplatin-induced apoptotic model, a JNK agonist was used to activate JNK. The results demonstrate that a JNK agonist can reverse the expression of p53 inhibited by ginsenoside Rh1. Moreover, it can reverse the expression levels of the apoptotic proteins Bax, caspase-3, and kidney injury biomarker NGAL inhibited by ginsenoside Rh1. Therefore, the apoptosis of HK-2 cells via ginsenoside Rh1 might be inhibited through the JNK/p53 pathway.

In conclusion, ginsenoside Rh1 was found to alleviate HK-2 apoptosis in a cisplatin-induced injury model by inhibiting ROS production and the JNK/p53 pathway. Ginsenoside Rh1 might therefore be a promising drug for the alleviation of cisplatin-induced nephrotoxicity in malignant patients.

## Data Availability

All data are presented in the manuscript. Datasets used and/or analyzed in this study are available from the corresponding author upon reasonable request.
